# Lower Expression of *SLC27A1* Enhances Intramuscular Fat Deposition in Chicken via Down-Regulated Fatty Acid Oxidation Mediated by *CPT1A*

**DOI:** 10.3389/fphys.2017.00449

**Published:** 2017-06-29

**Authors:** Fengfang Qiu, Liang Xie, Jing-e Ma, Wen Luo, Li Zhang, Zhe Chao, Shaohao Chen, Qinghua Nie, Zhemin Lin, Xiquan Zhang

**Affiliations:** ^1^Guangdong Provincial Key Lab of Agro-Animal Genomics and Molecular Breeding, and Key Lab of Chicken Genetics, Breeding and Reproduction, Ministry of Agriculture, College of Animal Science, South China Agricultural UniversityGuangzhou, China; ^2^School of Chemistry, Biology and Material Science, East China University of TechnologyNanchang, China; ^3^Department of Poultry Science, Institute of Animal Science and Veterinary, Hainan Academy of Agricultural SciencesHaikou, China

**Keywords:** *SLC27A1*, Intramuscular fat, RNA-sequencing, lipid catabolism, fatty acid oxidation, *CPT1A*

## Abstract

Intramuscular fat (IMF) is recognized as the predominant factor affecting meat quality due to its positive correlation with tenderness, juiciness, and flavor. Chicken IMF deposition depends on the balance among lipid synthesis, transport, uptake, and subsequent metabolism, involving a lot of genes and pathways, however, its precise molecular mechanisms remain poorly understood. In the present study, the breast muscle tissue of female Wenchang chickens (WC) (higher IMF content, 1.24 in D120 and 1.62 in D180) and female White Recessive Rock chickens (WRR; lower IMF content, 0.53 in D120 and 0.90 in D180) were subjected to RNA-sequencing (RNA-seq) analysis. Results showed that many genes related to lipid catabolism, such as *SLC27A1, LPL, ABCA1*, and *CPT1A* were down-regulated in WC chickens, and these genes were involved in the PPAR signaling pathway and formed an IPA® network related to lipid metabolism. Furthermore, *SLC27A1* was more down-regulated in WRR.D180.B than in WRR.D120.B. Decreased cellular triglyceride (TG) and up-regulated *CPT1A* were observed in the *SLC27A1* overexpression QM-7 cells, and increased cellular triglyceride (TG) and down-regulated *CPT1A* were observed in the *SLC27A1* knockdown QM-7 cells. These results suggest that lower lipid catabolism exists in WC chickens but not in WRR chickens, and lower expression of *SLC27A1* facilitate IMF deposition in chicken *via* down-regulated fatty acid oxidation mediated by *CPT1A*. These findings indicate that reduced lipid catabolism, rather than increased lipid anabolism, contributes to chicken IMF deposition.

## Introduction

Meat products are important components of human food. In the last several decades, great progress has been made in meat quantity by genetic selection for growth rate and meat yield, however, higher growth rate also induced larger fiber diameters, higher proportion glycolytic fibers, and lower intramuscular fat, which seriously deteriorated the quality of meat (Dransfield and Sosnicki, 1999; Du et al., 2010; Petracci and Cavani, 2012). It is an ongoing challenge to improve meat quality meanwhile maintain growth rate.

Meat quality is affected by many factors, among which intramuscular fat (IMF) is predominant. IMF refers to the amount of fat within muscles, including those localized in the epimysium, perimysium, and endomysium (Fernandez et al., 1999a). Unlike adipose tissue, in which the major lipid category is triglyceride (TG, >90%), a significant proportion of IMF is phospholipid. IMF has a plentiful polyunsaturated fatty acid (PUFA) composition and content, such as linoleic acid (18:2n-6), a-linolenic acid (18:3n-3) and arachidonic acid (20:4n-6; Wood et al., 2008). These PUFAs are readily oxidized by heating, producing volatile components such as 2,4-Decadienal which improves the flavor of meat (Calkins and Hodgen, 2007). Lots of studies have shown that IMF content was positively correlated with flavor, juiciness, and tenderness (Fernandez et al., 1999a,b; Chartrin et al., 2006; Gao and Zhao, 2009; Cannata et al., 2010; Hocquette et al., 2010; Madeira et al., 2013).

**Figure 1:**
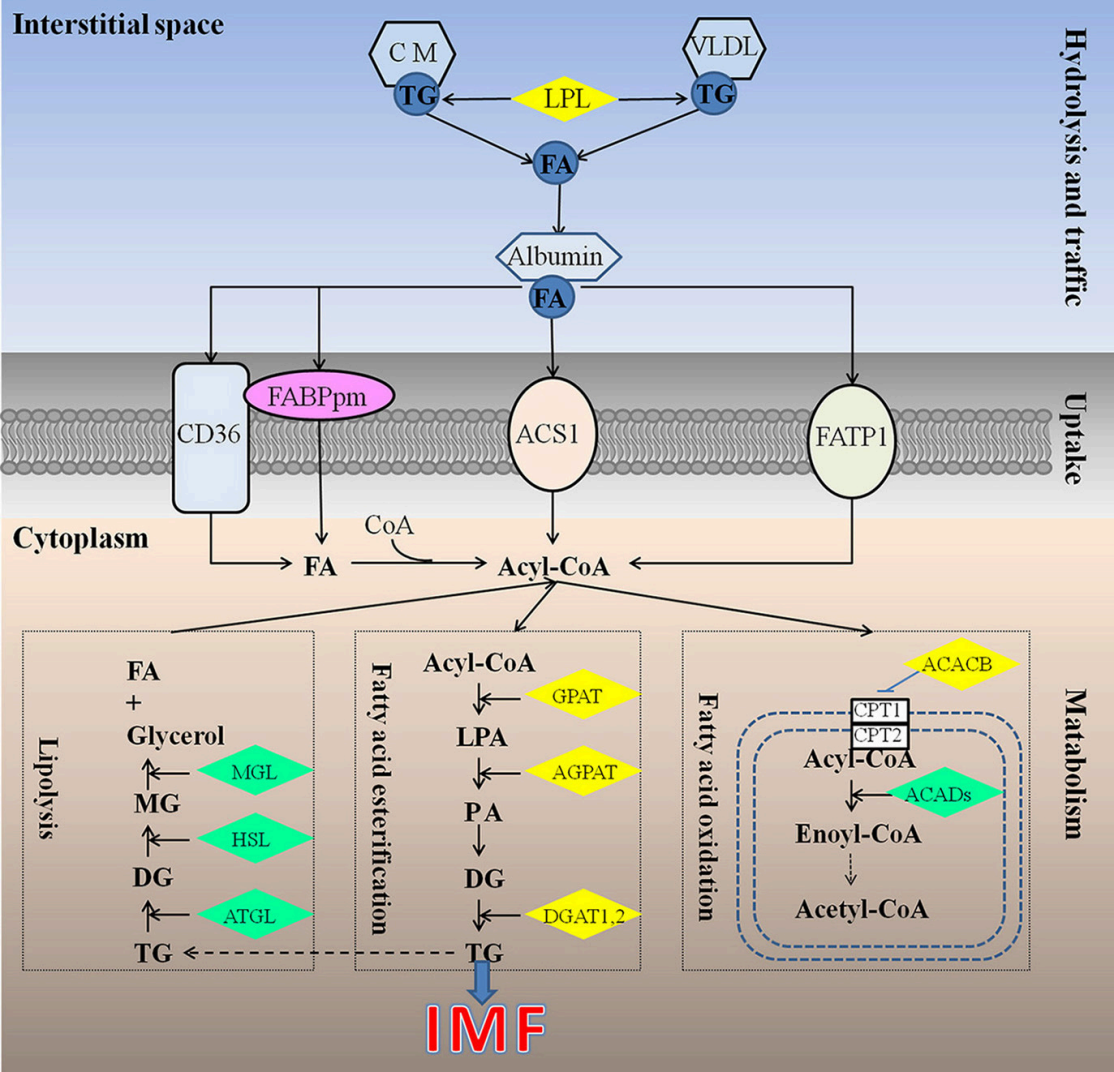
Underlying mechanism for IMF deposition in chickens. Hydrolysis and traffic: Both endogenous lipids synthesized by liver and exogenous lipids uptake from diet were hydrolyzed by LPL (Griffin et al., 1987), to produce FA. FA bound with albumin immediately, and be transported to muscles or other tissues (Schwenk et al., 2010). Uptake: it is generally recognized that fatty acids cross the cell membrane via a protein-mediated mechanism rather than by passive diffusion, and 4 proteins be identified responsible for fatty acids uptake, such as FABPpm, CD36, FATP1, and ACS1, could act, either alone or together, to enhance the fatty acid influx events (Glatz et al., 2010). Metabolism: IMF accumulation depends on the balance among fatty acids esterification, lipolysis, and oxidation (Jeong et al., 2012). ACACB, acetyl-CoA carboxylase beta; ACADs, acyl-CoA dehydrogenases; ACS1, Acyl-CoA synthetase; AGPAT, 1-acylglycerol-3-phosphate O-acyltransferase; ATGL, triglyceride lipase; CD36, Fatty acid translocase; CM, chylomicron; CPT1, carnitine palmitoyltransferase 1; CPT2, carnitine palmitoyltransferase 2; DG, diacylglycerol; DGAT, diacylglycerol acyltransferase; FA, fatty acid; FABPpm, Plasm membrane Fatty acid binding protein; FATP1, Fatty acid transporter 1; GPAT, glycerol-3-phosphate acyltransferases; HSL, hormone sensitive lipase; LPA, lysophosphatidic acid; LPL, lipoprotein lipase; MG, monoacylglycerol; MGL, monoacylglycerol lipase; PA, phosphatidic acid; TG, triglyceride; VLDL, very low density lipoprotein.

Different from mammals, in which *de novo* synthesis of fatty acids mainly occurs in adipocytes, the chicken synthesize its fatty acids predominantly in the liver (Leveille, 1969; Vernon et al., 1999), similar to fish (Rollin et al., 2003), and then exported to other tissues including both muscle and adipose tissue by the peripheral vascular system. Therefore, chicken IMF accumulation is dependent on the transport and uptake of blood lipids as well as lipogenesis subsequently in muscle rather than *de novo* fatty acids synthesis (Griffin et al., 1987). Previous studies have identified about 20 quantitative trait loci (QTL) related to chicken IMF, which are mainly located on chromosomes 1, 2, 5, 23 (Jennen et al., 2005; D'Andre et al., 2010; Ye et al., 2010; Jia et al., 2012; Liu et al., 2013; Nassar et al., 2013; Sun et al., 2013; Zhang T. et al., 2015). Otherwise, a large number of genes including *GPAT1, ACC, CD36, AGPAT1*, and *DGAT2* (Jeong et al., 2012), *FABP* (Ye et al., 2010; Serao et al., 2011), *LPL* (Zhang X. D. et al., 2015), *DGAT1* (Li et al., 2013) were recognized as candidate genes for IMF, but their molecular mechanisms affecting IMF are still unclear. Obviously, the mechanism underlying chicken IMF deposition is very complicated, involving a lot of genes and metabolic pathways (Figure 1).

Although, previous studies have analyzed the transcriptome of chicken breast muscle (Cui et al., 2012) and liver (Bourneuf et al., 2006) utilizing microarrays, and identified some potential candidate genes and pathways that influence chicken breast muscle IMF deposition, no further validation has been performed. Therefore, the precise molecular mechanisms underlying chicken IMF deposition have not been fully elucidated so far. In the present study, female breast muscle of Wenchang chicken (WC, Chinese native breed, high IMF content) and Recessive White Rock chicken (WRR, imported and fast-growing breed, low IMF content) were subjected to RNA-sequencing (RNA-seq) analyses. Results showed that chicken IMF deposition is attributable to some key genes and pathways related to lipid catabolism rather than lipid anabolism, and the higher IMF deposition might resulted from lower lipid oxidation.

## Materials and methods

### Ethics statement

All animal procedures were authorized by the Animal Care Committee of South China Agricultural University (Guangzhou, China). Animals involved in the present study were sacrificed as necessary to ameliorate their suffering.

### Animals and sample collection

In the present study, WC chickens were collected from Longquan Wenchang Chicken Industrial co., LTD (Wenchang, Hainan, China), and WRR chickens were collected from Guangdong Wen's Food Group, LTD (Xinxing, Guangdong, China). Birds were raised up to 120 d of age (D120) or 180 d of age (D180), six female birds of similar weight from each breed per age were sacrificed for muscle sample collection. The center of right breast muscle (B) was excised, divided into three parts for RNA-seq, qRT-PCR, and Oil Red O staining respectively, snap-frozen in liquid nitrogen immediately and stored long-term at −80°C. The whole left breast muscle was excised for IMF content measurement and stored at −30°C.

### Oil red O staining and IMF content measurement

Frozen tissues were sectioned using a Leica Kryostat (Leica CM3050S, Leica instrument GmbH, Germany), fixed 30 min with 10% paraformaldehyde, and incubated 15 min at room temperature with Oil Red O, then visualized with light microscopy.

IMF contents were measured in a Soxhlet apparatus (Soxtherm, German), according to the description elsewhere (Zerehdaran et al., 2004; Cui et al., 2012). The data are represented as percentages of the wet weight of the muscle.

### Total RNA extraction and cDNA synthesis

Total RNA was extracted from muscle samples or cells with RNAiso reagent (Takara, Otsu, Japan) and treated with DNase I (Takara). The integrity and concentration of RNA were assessed by 1.2% denatured gel electrophoresis and NanoDrop 2000c instrument (Thermo, Waltham, MA, USA). cDNA synthesis was carried out using a PrimeScript RT reagent Kit (Perfect Real Time) (Takara) according to the manufacturer's instruction.

### RNA-Seq analyses

For each breed at each age, two birds were selected randomly from the total of six birds for RNA-seq respectively. So eight muscle samples in total were subjected to RNA-seq analysis, four WC chicken samples including WC.D120.B-1, WC.D120.B-3, WC.D180.B-2, and WC.D180.B-3, and four WRR chicken samples including WRR.D120.B-4, WRR.D120.B-6, WRR.D180.B-2, and WRR.D180.B-5.

After the total RNA extraction and DNase I treatment, magnetic beads with Oligo (dT) were used for mRNA enrichment. The enriched mRNA was fragmented, and used as templates for synthesizing cDNA. Short cDNA fragments were purified, end-repaired, tailed with single nucleotide adenine, and then connected with adapters. With agarose gel electrophoresis, suitable fragments were amplified with PCR. Subsequently the sample library was assessed with an Agilent 2100 Bioanaylzer and ABI StepOnePlus Real-Time PCR System for quantity and quality. Finally, the library was sequenced using Illumina HiSeq™ 2000 (BGI, China).

Primary sequencing data that produced by Illumina HiSeq™ 2000, called raw reads, were subjected to quality control (QC) to determine whether a resequencing step was needed. After QC, raw reads were filtered, and clean reads were aligned to reference sequences with *SOAPaligner/SOAP2* (Li et al., 2009). Then the alignment data were utilized to calculate distribution and coverage of reads on reference genes. Next, we proceeded with gene expression analysis, which included gene expression level and differential expression gene (DEG). Furthermore, we performed Gene Ontology (GO) enrichment analysis (Ashburner et al., 2000; Young et al., 2010), KEGG (Kyoto Encyclopedia of Genes and Genomes, http://www.genome.jp/kegg/) Pathway enrichment analysis (Altermann and Klaenhammer, 2005; Kanehisa et al., 2008) and Ingenuity® Pathway Analysis (IPA®, http://www.ingenuity.com/) of DEGs.

### Quantitative RT-PCR analysis

The primers for qRT-PCR were designed by Primer premier 5 software. The forward (F) and reverse (R) primer of each gene were derived from different exons, and the size of each PCR product was about 150 ~ 250 bp. qRT-PCR was carried out in a Bio-rad CFX96 Real-Time Detection system (Bio-rad, Hercules, CA, USA) employing KAPA SYBR FAST q-PCR Kit (KAPA Biosystems, Wobrun, MA, USA) according to the manufacturer's instruction. The 2^−(ΔΔCt)^ formula was used to quantify the relative gene expression with *GAPDH* as a reference gene (Vandesompele et al., 2002).

### Plasmids construction and siRNA oligonucleotides synthesis

*pcDNA-3.1(*+*)-SLC27A1* expression vector. The *SLC27A1* coding sequence containing 1941 base pairs was amplified from chicken breast muscle cDNA, and the specific primers as follows: 5′-cttaagcttatgcagcccgtgggggtgt-3′ and 5′-gcagaattctcataaggcgactttcccggagca-3′. The PCR product was cloned into the pcDNA-3.1(+) vector (Promega, Madison, WI, USA) using restriction enzymes *EcoR*I and *Hind*III.

SiRNA oligonucleotides and negative control (NC) for gene interference were purchased from GenePharma (GenePharma, Suzhou, China).

### Cell culture

The QM-7 myoblasts were incubated in a humidified atmosphere containing 5% CO_2_ in M199 medium containing 10% fetal bovine serum (FBS), 10% tryptose phosphate broth, 0.1% penicillin, and 0.1% streptomycin (growth medium). Cells were incubated in this growth medium for 48 h, the density reached to 80 ~ 90%. Then the cells were induced to differentiate by lowering FBS concentration to 1% (differentiation medium) for another 48 h, reaching 80 ~ 90% preconfluent. Then the preconfluent myoblasts were used for subsequent transfection experiments.

### Transfection experiment

The plasmids *pcDNA3.1(*+*)-SLC27A1* and *pcDNA3.1(*+*)-EGFP*, or gga-1220 (siRNA) and NC, were transfected in QM-7 cells which incubated in 6-well plates using Lipofectamine® 3000 reagent (Invitrogen) following the manufacturer's guidelines. After that, cells were incubated and differentiated for 48 h, and reaching 80 ~ 90% confluent, then used for subsequent experiments.

### Measurement of cellular TG and FFA

Cells pretreated as described above were incubated further for 16 h at 37°C in serum free M199 medium containing 0.30 mM palmitate (Sigma, USA) bound to 1% bovine serum albumin (BSA, Sigma, USA) and 0.05 mM L-carnitine (Sigma, USA). After that, the cells were washed, collected and diluted with phosphate-buffered saline (PBS, pH 7.2–7.4). Cellular TG and free fatty acid (FFA) concentrations were measured using Chicken Triglyceride (TG) and FFA ELISA Kits (Jiyinmei Biological Technology Co., LTD, Wuhan, China), according to the manufacturer's guidelines.

### Statistical analyses

Data are presented as means ± S.E.M based on at least three replicates for each treatment. A one-way ANOVA was used to determine statistical significance, where *P* < 0.05 was considered significant differences.

## Results

### IMF content of WC chicken and WRR chicken

**Figure 2:**
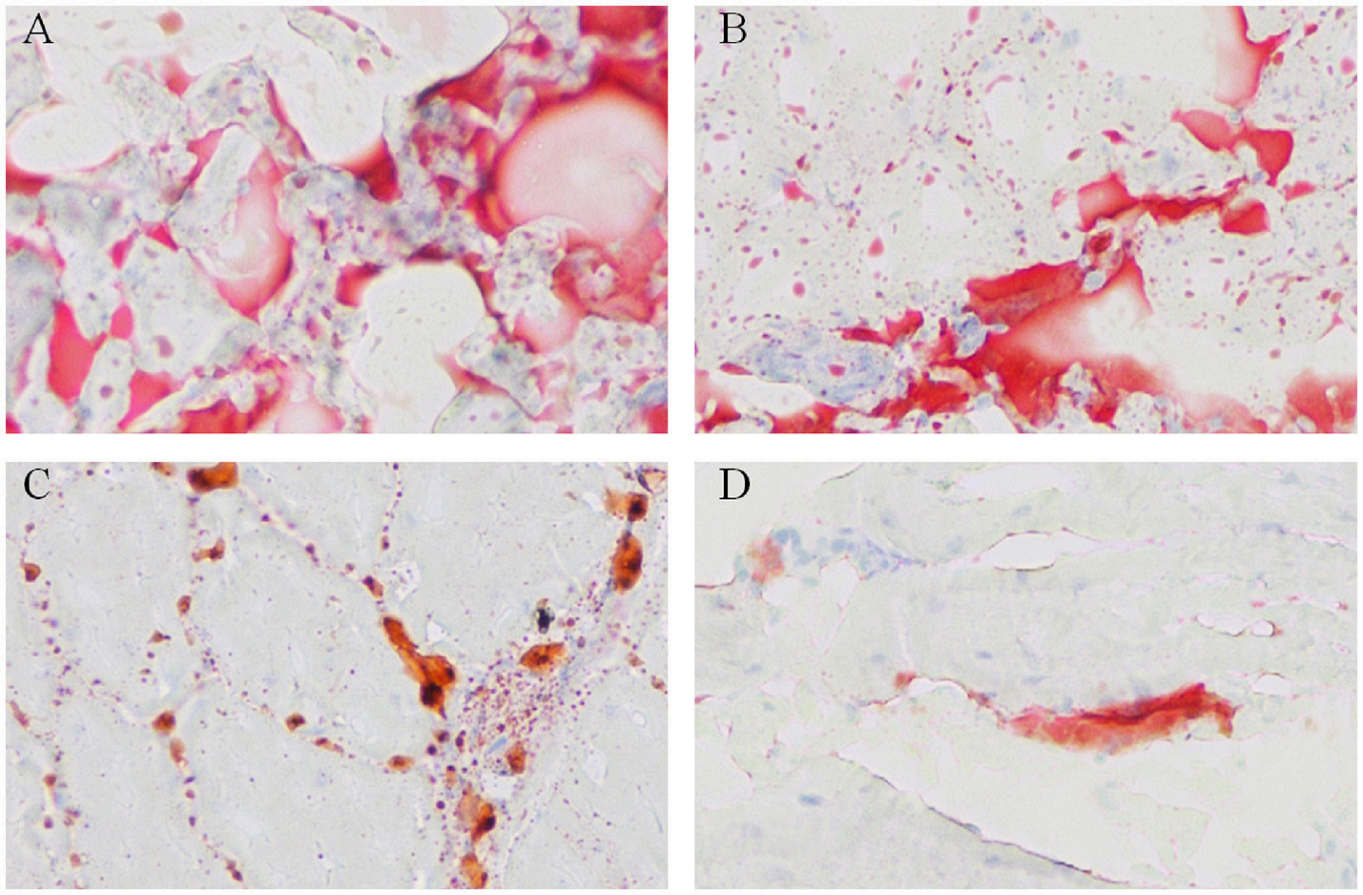
Oil Red O staining for neutral lipids in breast muscle of WC and WRR chickens (200X). Breast muscle samples were paraformaldehyde fixed and incubated with Oil Red O and visualized by light microscopy. **(A)** Lipids in WC.D180.B. **(B)** Lipids in WC.D120.B. **(C)** Lipids in WRR.D180.B. **(D)** Lipids in WRR.D120.B. The picture presented is representative of three independent experiments.

To visualize the difference of IMF deposition between breeds and within breeds, breast muscle samples were subjected to Oil Red O staining. Between the two breeds, the density and size of lipids in WC.D120.B (Figure 2A) and WC.D180.B (Figure 2B) were larger than those of in WRR.D120.B (Figure 2C) and WRR.D180.B (Figure 2D). Within breed, the density and size of lipids in D180 muscles were larger than that of in D120 muscles. To further quantify the difference of IMF deposition in chickens, we measured the IMF content of breast muscles using Soxhlet apparatus and the data were showed in Table 1. IMF content of WC muscles were significantly higher than that of WRR muscles (*P* < 0.01), 1.24 vs. 0.53 in D120 and 1.62 vs. 0.90 in D180, respectively. Within breed, the IMF content in D180 muscles were significantly higher than that in D120 muscles (*P* < 0.01).

**Table 1:** IMF content of WC and WRR (WET, %).

** 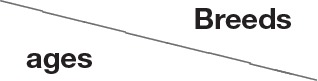 **	**WC**	**WRR**
D120	1.24 ± 0.09^*A*^	0.53 ± 0.11^*B*^
D180	1.62 ± 0.14^A^^*^	0.90 ± 0.05^B^^**^

*Data are presented as means ± S.E.M (n = 6), different capital superscripts in each row indicate extremely significant differences (P < 0.01)*,

* *in each column means significant differences (P < 0.05)*,

** *in each column means extremely significant differences (P < 0.01)*.

*WC, Wenchang chicken; WRR, White Recessive Rock chicken*.

### Transcriptome assembly and statistics of RNA-Seq

To identify the underlying molecular mechanism responsible for chicken IMF deposition, the transcriptome of breast muscle of WC chicken and WRR chicken at different ages were analyzed with RNA-seq. Two birds per breed of each age were selected to produce cDNA libraries for RNA-seq separately. The clean reads of each sample were over 20 million, the expressed genes ranged from 16,202 to 17,838, and a little higher in WRR samples than in WC samples (Table 2).

**Table 2:** Statistics of RNA-seq data.

**Samples**	**Clean reads**	**Genome map rate (%)**	**Gene map rate (%)**	**Expressed genes**
WC.D120.B-1	20566818	63.09	64.11	16202
WC.D120.B-3	21126204	63.85	64.86	16475
WC.D180.B-2	23644072	65.81	60.99	16821
WC.D180.B-3	24164920	64.18	67.30	17136
WRR.D120.B-4	25413150	62.87	64.02	17349
WRR.D120.B-6	25407212	64.26	64.48	17340
WRR.D180.B-2	23458948	66.03	63.38	17240
WRR.D180.B-5	23346692	65.54	66.86	17838

*WC, Wenchang chicken; WRR, White Recessive Rock chicken; B, breast muscle*.

### Differentially expressed genes (DEGs)

**Figure 3:**
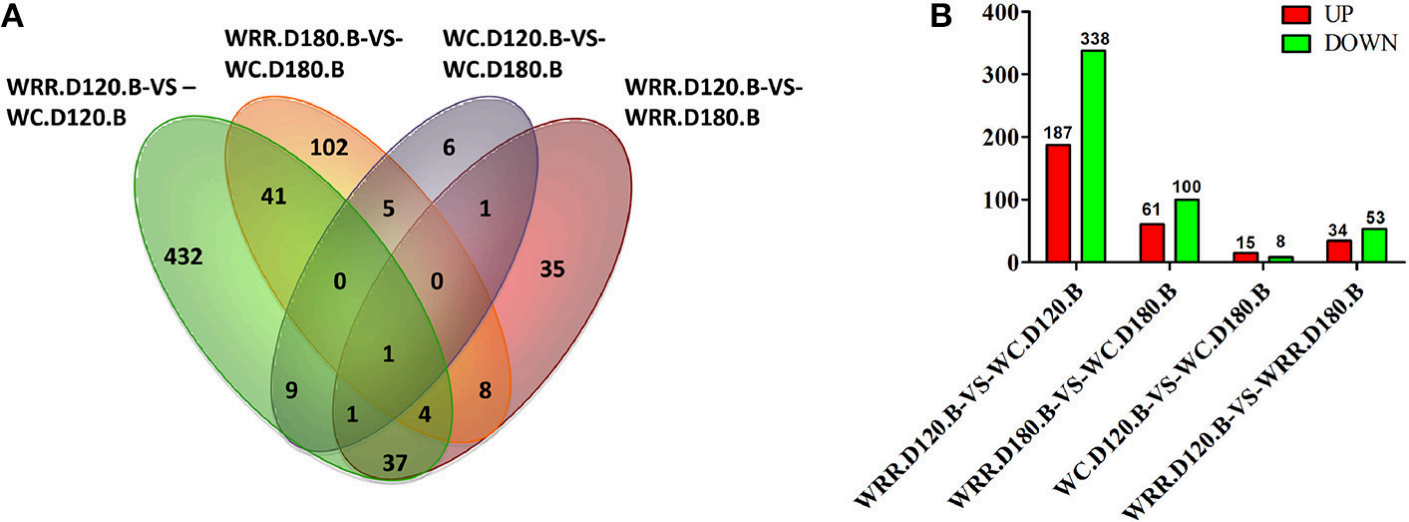
Differentially expressed genes among four comparisons. **(A)** Differentially expressed genes that are unique or shared among four comparisons. **(B)** Differentially expressed genes that are up-regulated expression (red) or down-regulated expression (green) in the latter of each contrast. WC, Wenchang chicken; WRR, White Recessive Rock chicken; B, breast muscle.

Generally, false discovery rate (FDR) < 0.05 and two-fold difference together was regarded as differentially expressed. In the present study, there were 525, 161, 23, 87 DEGs detected in the comparisons of WRR.D120.B-VS-WC.D120.B, WRR.D180.B-VS-WC.D180.B, WC.D120.B-VS-WC.D180.B, and WRR.D120.B-VS-WRR.D180.B, respectively (Figure 3).

Of the shared DEGs among four comparisons, *FBXO32* was the most common gene shared by all four comparisons, while *PPP3CA* was the common gene in WRR.D120.B-VS-WC.D120.B, WC.D120.B-VS-WC.D180.B, and WRR.D120.B-VS-WRR.D180.B. In addition, a total of 46 DEGs were shared by WRR.D120.B-VS-WC.D120.B and WRR.D180.B-VS-WC.D180.B, of which 12 were up-regulated in WC chicken, the rest were up-regulated in WRR chicken. These DEGs may be correlated with the different development properties of breast muscle between breeds. Otherwise, *MUSTN1* and *PPP3CA* were the other two common genes shared by WC.D120.B-VS-WC.D180.B and WRR.D120.B-VS-WRR.D180.B, which might be involved in regulating the temporal characteristics of breast muscle development. The shared DEGs were presented in Supplementary Table 1.

### Quantitative real-time PCR verification of DEGs

**Figure 4:**
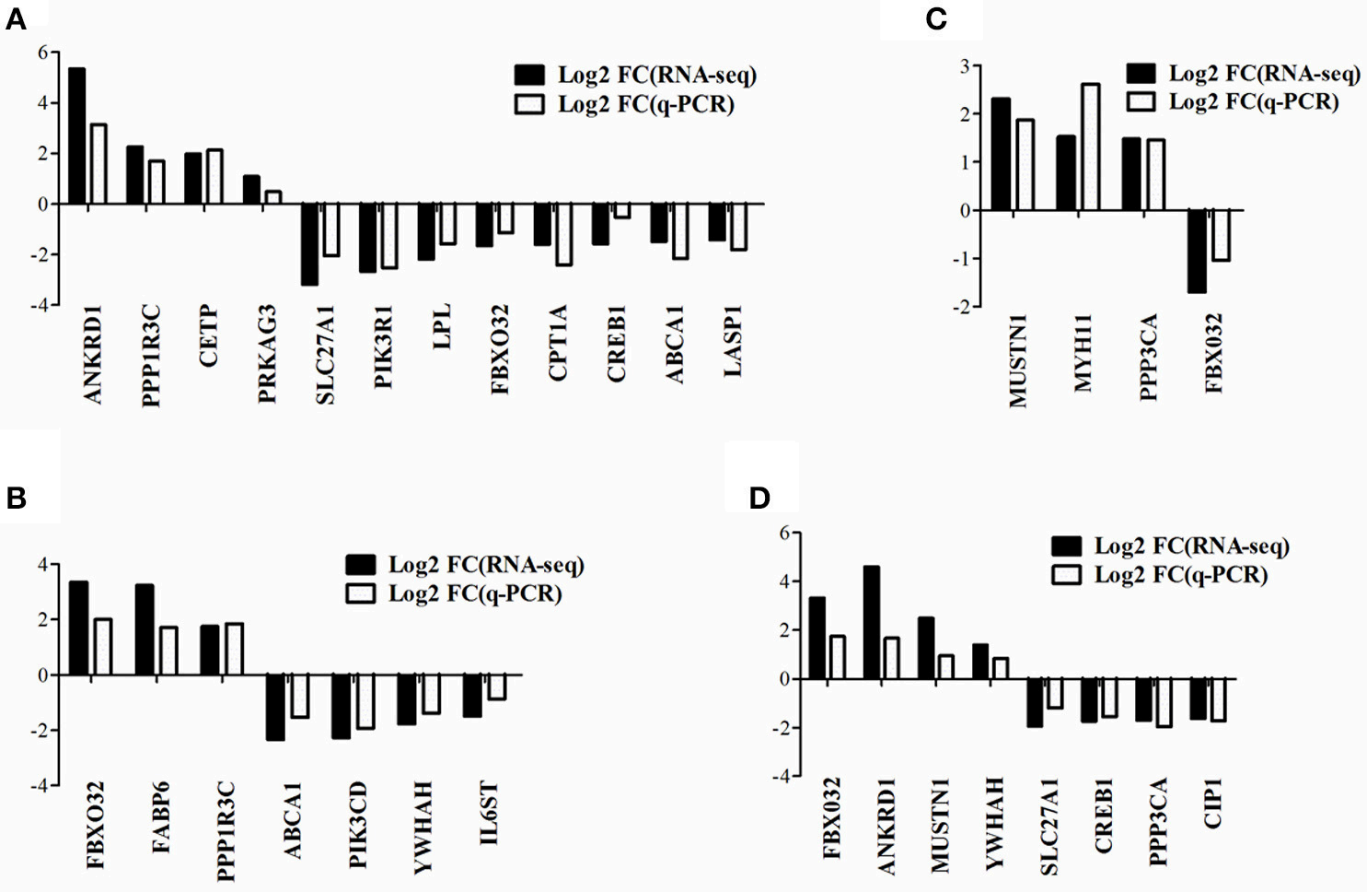
Quantitative real-time PCR verification of DEGs detected from RNA-seq. The data presented are representative of three independent experiments performed at least three biological replicates; mean ± S.E.M. **(A)** DEGs in WRR.D120.B-VS-WC.D120.B. **(B)** DEGs in WRR.D180.B-VS-WC.D180.B. **(C)** DEGs in WC.D120.B-VS-WC.D180.B. **(D)** DEGs in WRR.D120.B-VS- WRR.D180.B.

To verify the gene expression pattern of DEGs detected by RNA-seq, a total of 20 genes were selected randomly to perform quantitative real-time PCR experiments and the primers were listed in Supplementary Table 2. The gene expression pattern of quantitative real-time PCR were generally accordant with that of RNA-seq, although different in fold changes (Figure 4), which indicated that our RNA-seq data were reliable.

### GO terms for DEGs

**Figure 5:**
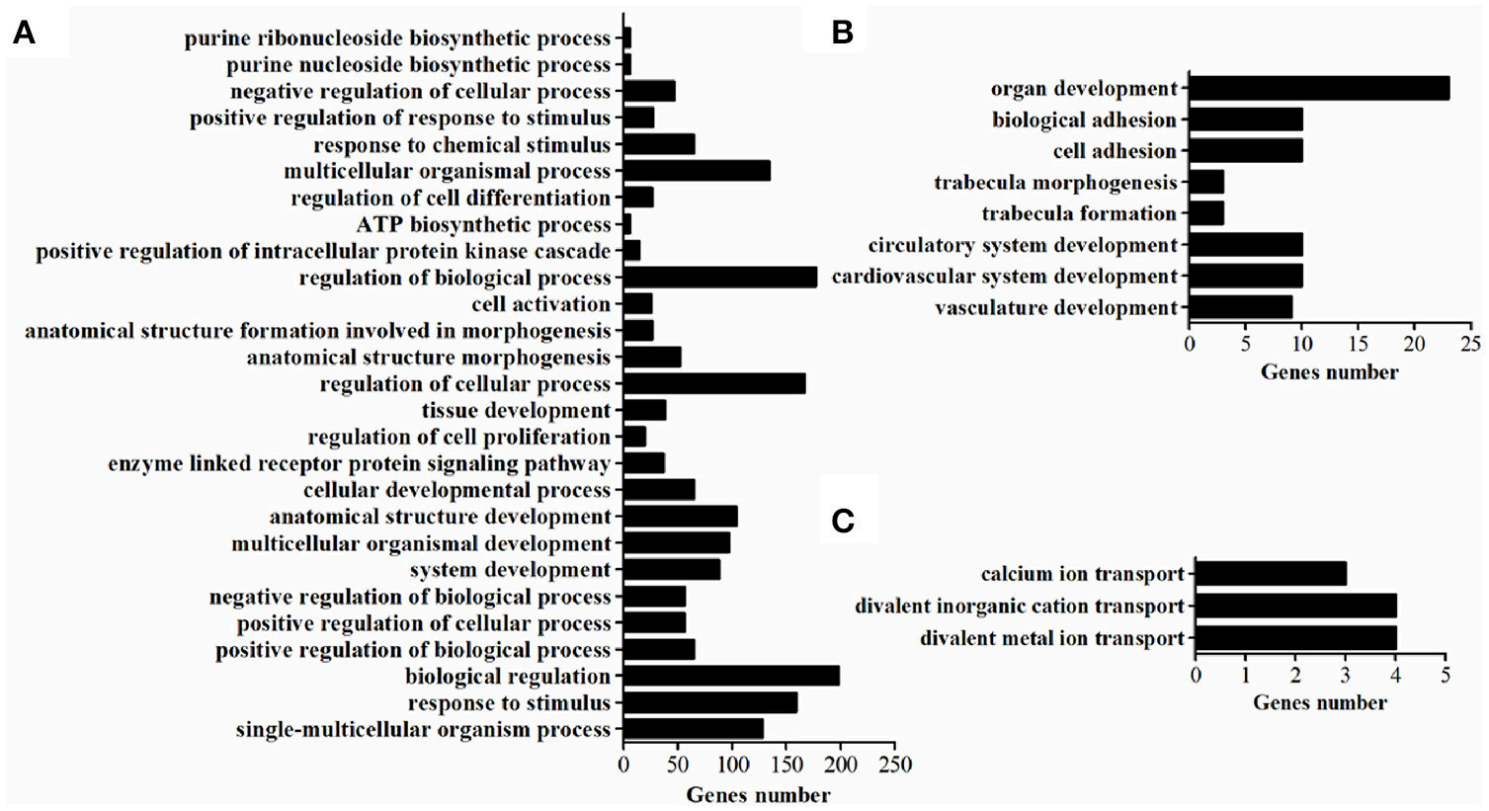
The enriched biological processes of DEGs. **(A)** Biological processes enriched in DEGs in WRR.D120.B-VS-WC.D120.B. **(B)** Biological processes enriched in DEGs in WRR.D180.B-VS- WC.D180.B. **(C)** Biological processes enriched in DEGs in WC.D120.B-VS-WC.D180.B.

GO-term analysis was used to investigate the function of DEGs. In the present study, the enriched GO-terms (Corrected *P* < 0.05) in the ontology classification “biological process” were represented in Figure 5. Between breeds, the enriched biological process mainly including multicellular organismal process, response to stimulus, biological regulation, regulation of biological process, regulation of cellular process, cellular developmental process, tissue development. The enriched biological process within WC chicken mainly focused on ion transport (Figure 5), no biological process was enriched within WRR chicken.

### Key DEGs associated with chicken IMF deposition

Based on GO function annotation, DEGs related to lipid metabolism would be attributable to chicken IMF deposition. Total of 33 (between breeds) and seven (between ages) DEGs were associated with IMF deposition (Supplementary Table 3). There were 24 DEGs down-regulated in WC chicken, including several well-known candidate genes for lipid metabolism, such as *ABCA1* (Santamarina-Fojo et al., 2001), *ACACB* (Xu et al., 2014), *CPT1A* (Akkaoui et al., 2009), *LPL* (Claire et al., 2013; Zhang X. D. et al., 2015), and *SLC27A1* (Sebastian et al., 2009; Guitart et al., 2014), of which *SLC27A1* was also more down-regulated in WRR.D180.B than in WRR.D120.B. In other words, *SLC27A1* were generally lower expressed in the higher IMF muscles.

### Pathways for DEGs

**Figure 6:**
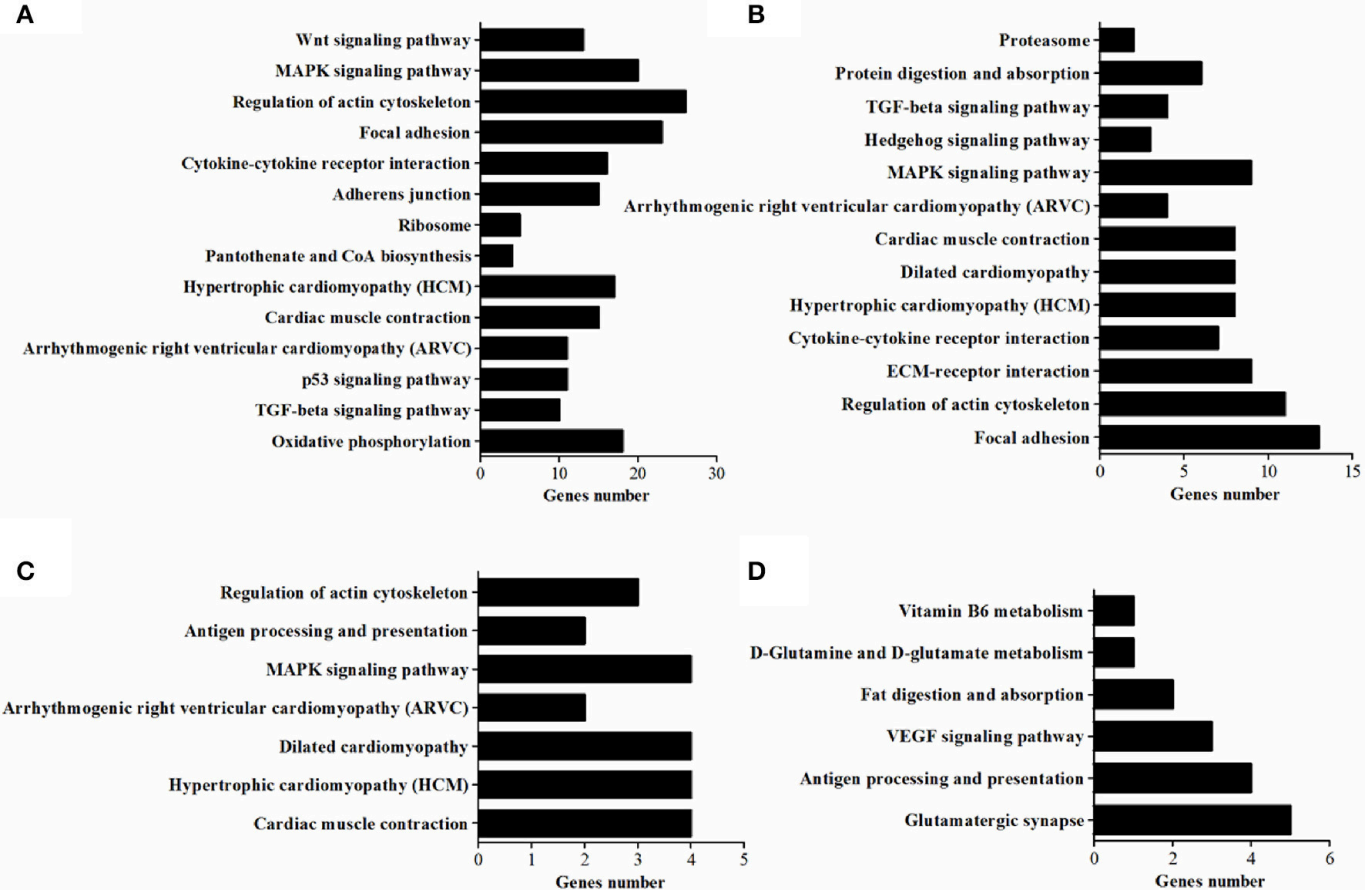
The enriched KEGG pathways of DEGs. **(A)** Pathways enriched in DEGs in WRR.D120.B- VS-WC.D120.B. **(B)** Pathways enriched in DEGs in WRR.D180.B-VS- WC.D180.B. **(C)** Pathways enriched in DEGs in WC.D120.B-VS-WC.D180.B. **(D)** Pathways enriched in DEGs in WRR.D120.B-VS-WRR.D180.B.

**Figure 7:**
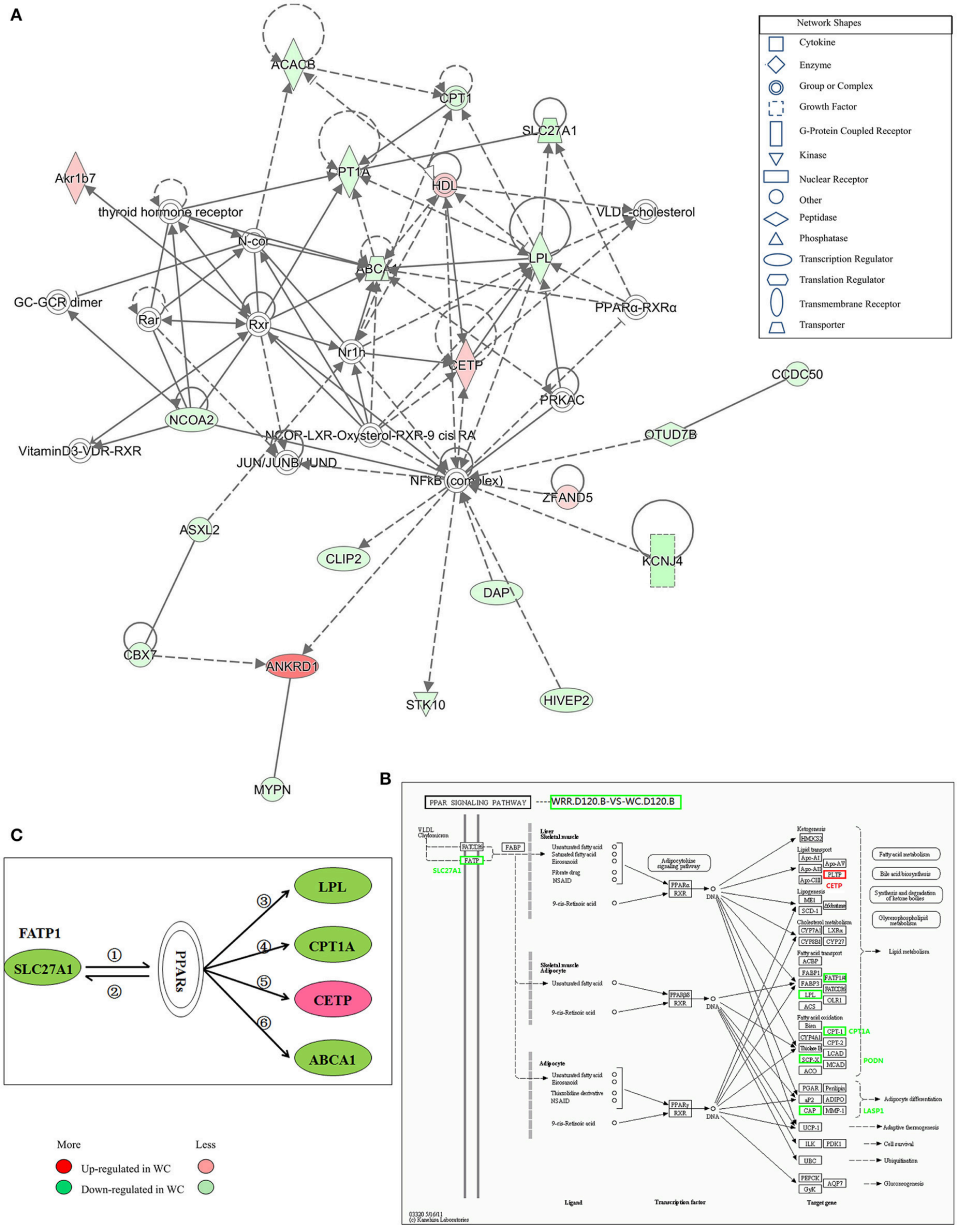
Gene interaction network related to lipid metabolism of DEGs between WC and WRR chickens. **(A)** Functional gene interaction networks was identified by Ingenuity Pathway Analysis (IPA®) software. This network shows direct gene interactions mainly in WRR.D120.B-VS-WC.D120.B related to lipid metabolism, molecular transport, small molecule biochemistry. **(B)** DEGs *SLC27A1, LPL, CPT1A, CETP* in WRR.D120.B-VS-WC.D120.B involved in PPAR signaling pathway. **(C)** Proposed network for *SLC27A1* to regulate chicken lipid metabolism based on GO annotation, KEGG pathway and Ingenuity Pathway Analysis (IPA®). ① *SLC27A1* overexpression increases fatty acids afflux, which offers ligands (Forman et al., 1995) for binding and activating PPARs; ② expression of FATP genes (*SLC27A1*) involves both PPARα and –γ (Motojima et al., 1998); ③ PPAR control triglyceride metabolism by transcriptional control the expression of *LPL* (Auwerx et al., 1996); ④ Though binding to PPAR responsive element in *CPT1A*, PPAR promotes fatty acid oxidation (Mascaro et al., 1998; Song et al., 2010); ⑤ PPAR activation may inhibit plasma CETP activity (Li and Chiang, 2009); ⑥ PPARs and LXRs are involved in the regulation of *ABCA1* expression (Chinetti et al., 2001). Genes colored in red are up-regulated expression in WC chickens, while genes colored in green are down-regulated expression in WC chickens. Color intensity correlates to the size of the fold change.

*In vivo*, lots of genes execute their biological function concertedly, and pathway enrichment analysis can reveal the main biochemical metabolism process and signal transduction pathways in which DEGs are involved. In the present study, pathways significantly enriched with DEGs were assessed by a hypergeometric test using R packages (*P* < 0.05, FDR adjusted). As presented in Figure 6, there were 14 (in D120) and 13 (in D180) pathways identified in DEGs between breeds, with eight being shared by two ages which included MAPK signaling, TGF-beta, cytokine-cytokine receptor interaction, focal adhesion, regulation of actin cytoskeleton, arrhythmogenic right ventricular cardiomyopathy (ARVC), cardiac muscle contraction, hypertrophic cardiomyopathy (HCM). Within breeds, between different ages, DEGs in WC chicken were enriched in MAPK signaling and regulation of actin cytoskeleton, while DEGs in WRR chicken were enriched in VEGF signaling and fat digestion and absorption (Figure 6). Similar results were obtained by previous study on Beijing-you chickens and Arbor Acres chickens using Agilent cDNA microarray (Cui et al., 2012). These results indicate that not only the pathways related to lipid metabolism (MAPK signaling; Kokta et al., 2004; Du et al., 2010), but also those involved in cell-links [cytokine-cytokine receptor interaction (Ozaki and Leonard, 2002), focal adhesion (Petit and Thiery, 2000), regulation of actin cytoskeleton (Pollard, 2003)] and cell cycle (TGF-beta; Shi and Massague, 2003) could dedicate to the IMF deposition, for their crucial role in maintaining the morphology of tissue and cell growth and proliferation. In addition, key DEGs related to lipid metabolism include *CPT1A, CETP, LPL*, and *SLC27A1*, all being involved in PPAR signaling pathway (Figure 7B), although it did not reach up to statistical significance (*P* > 0.05).

### Gene networks for DEGs

IPA® was performed to investigate genes affecting lipid metabolism between breeds. A total of 16 networks were identified in WRR.D120.B-VS-WC.D120.B (Supplementary Table 4) to be associated with post-translational modification, organismal injury and abnormalities, cell-to-cell signaling and interaction, energy production, cancer and lipid metabolism. The DEGs *ABCA1, CPT1A, LPL*, and *SLC27A1*, which were down-regulated in WC.D120.B and involved in PPAR signaling pathway (Figure 7B), acted as important node gene and interacted in the network of lipid metabolism, molecular transport, small molecule biochemistry (Figure 7A). *CETP* was another important node gene in this network, but up-regulated in WC.D120.B.

In WRR.D180.B-VS-WC.D180.B, five networks were identified (Supplementary Table 4) and they were mainly related to connective tissue development and function, cancer, organismal injury and abnormalities, nerve system development and function, dermatological diseases and conditions.

### Proposed network for *SLC27A1* to regulate chicken IMF deposition

Based on the above results and previous study, a network for *SLC27A1* to regulate chicken IMF deposition was proposed (Figure 7C). In this proposed network, *SLC27A1* might regulate *LPL* (Auwerx et al., 1996), or *CPT1A* (Mascaro et al., 1998; Song et al., 2010), or *CETP* (Li and Chiang, 2009), or *ABCA1* (Chinetti et al., 2001), respectively or together via PPARs, and consequently regulate the lipid metabolism of WC chickens.

### *SLC27A1* overexpression decreased cellular TG and increased *CPT1A* mRNA

To further verify the role of *SLC27A1* in lipid metabolism and its proposed molecular mechanism (Figure 7C), we overexpressed *SLC27A1* in QM-7 cells. The primers for quantitative real-time PCR were presented in Supplementary Table 5.

**Figure 8:**
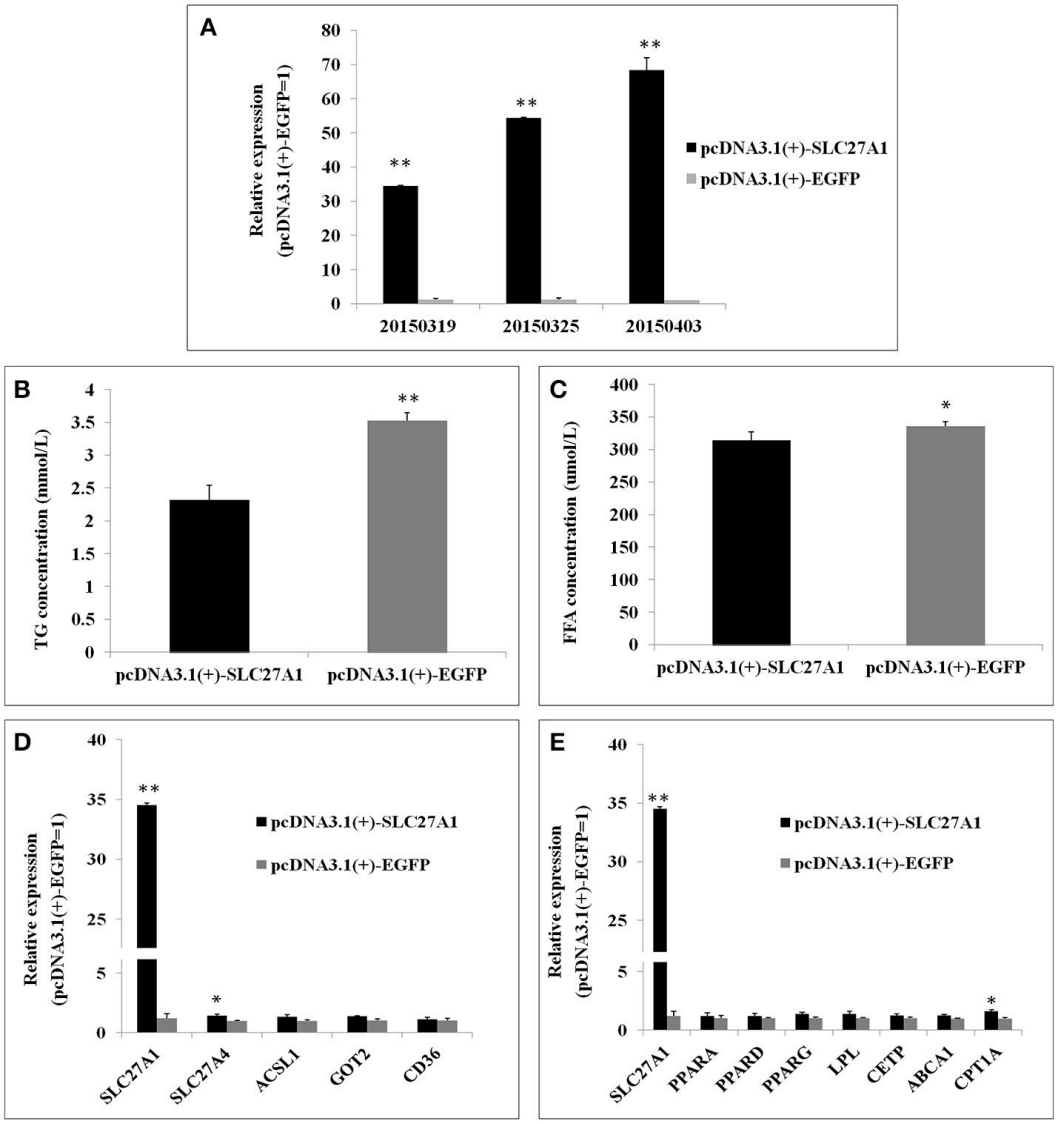
Effect of *SLC27A1* overexpression on lipid metabolism. **(A)** *SLC27A1* overexpression efficiency. **(B)** Palmitate incorporation into TGs. Data are the mean ± S.E.M of three experiments performed in triplicate. **(C)** Palmitic acid uptake. Data are the mean ± S.E.M of three experiments performed in triplicate. **(D)** Synergy or compensation effect exist among different fatty acid transports. The data presented are representative of three independent experiments; *n* = 3, mean ± S.E.M. **(E)** The regulative mechanism of lipid metabolism for *SLC27A1*. The data presented are representative of three independent experiments; *n* = 3, mean ± S.E.M. ^*^P < 0.05, *pcDNA3.1(*+*)-SLC27A1* vs. *pcDNA3.1(*+*)-EGFP*; ^**^P < 0.01, *pcDNA3.1(*+*)-SLC27A1* vs. *pcDNA3.1(*+*)-EGFP*.

Overexpression was achieved by transfection of QM-7 cells with *pcDNA3.1(*+*)-SLC27A1* containing chicken full-length *SLC27A1* cDNA. *SLC27A1* mRNA levels increased over 34-fold in *pcDNA3.1(*+*)-SLC27A1*-transfected cells compared with control *pcDNA3.1(*+*)-EGFP*-transfected cells (Figure 8A). To assess the function of the overexpressed *SLC27A1* in lipid metabolism, we measured the incorporation of palmitate into TG in QM-7 cells. Overexpressed *SLC27A1* resulted in extremely significant decreases of cellular TG concentration (2.32 ± 0.23 mm/L vs. 3.52 ± 0.12 mm/L, *P* < 0.01, Figure 8B), and significant decreases of cellular FFA concentration (335.35 ± 12.13 μm/L vs. 355.94 ± 6.32, *P* < 0.05, Figure 8C), compared with control cells.

To investigate whether synergy or compensation effect exists among different fatty acid transporters, we evaluated *SLC27A4, ACSL1, FABPpm/GOT2, FAT/CD36* mRNA levels in *pcDNA3.1(*+*)-SLC27A1*-transfected cells compared with control *pcDNA3.1(*+*)-EGFP*- transfected cells by quantitative real-time PCR (Figure 8D). *SLC27A1* overexpression resulted in the up-regulated for *SLC27A4* (*P* < 0.05), and no change was observed for *ACSL1, GOT2*, and *CD36*.

To investigate the molecular mechanism and biological pathway for *SLC27A1* affecting IMF deposition, we assessed the mRNA levels of PPARs and DEGs involved in PPAR signaling pathway in *pcDNA3.1(*+*)-SLC27A1*-transfected cells compared with control *pcDNA3.1(*+*)-EGFP*-transfected cells by quantitative real-time PCR (Figure 8E). Overexpressed *SLC27A1* resulted in the up-regulated for and *CPT1A* (*P* < 0.05), while no changes were observed for other genes.

### *SLC27A1* knockdown increased cellular TG and decreased *CPT1A* mRNA

**Figure 9:**
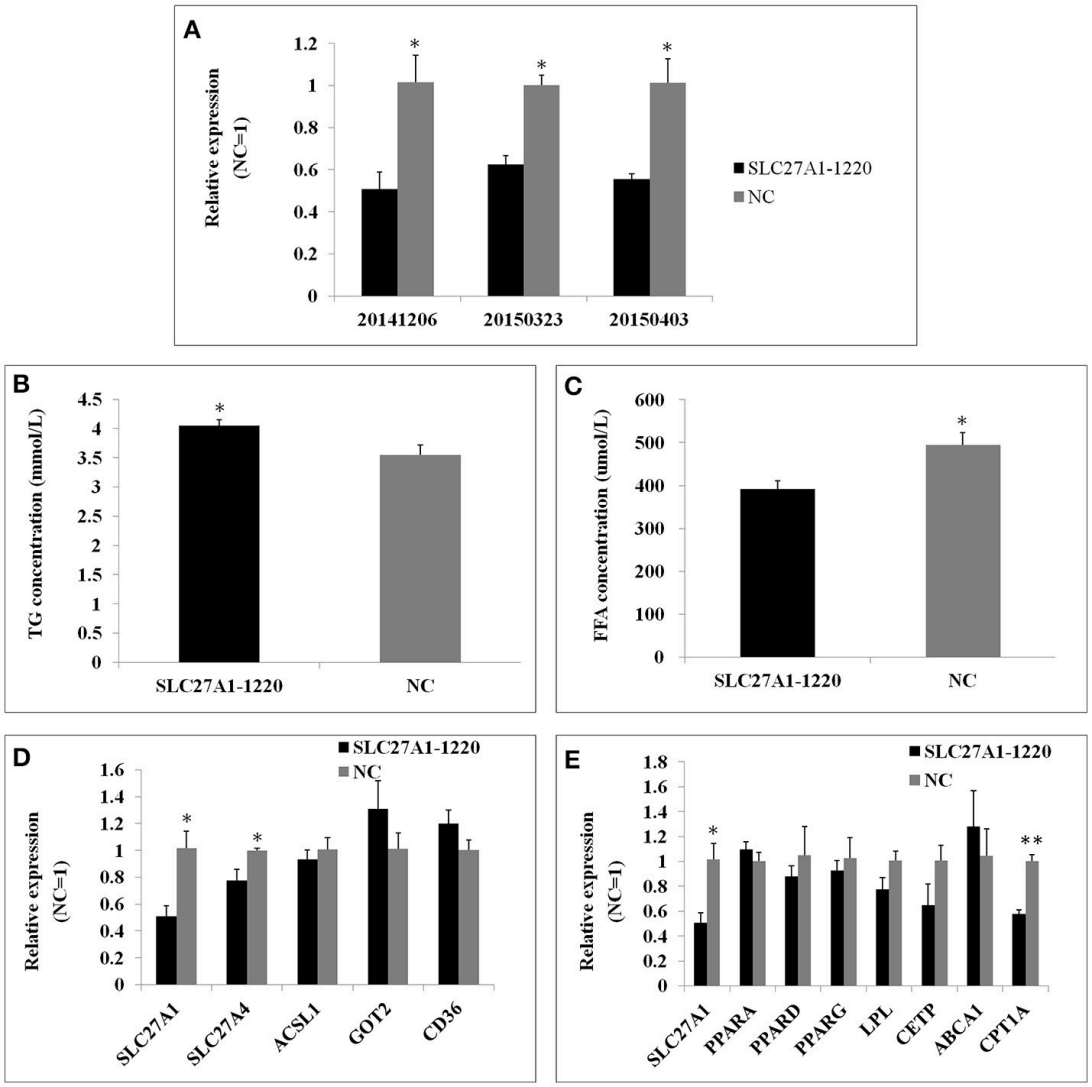
Effect of *SLC27A1* knockdown on lipid metabolism. **(A)** *SLC27A1* interference efficiency. **(B)** Palmitate incorporation into TGs. Data are the mean ± S.E.M of three experiments performed in triplicate. **(C)** Palmitic acid uptake. Data are the mean ± S.E.M of three experiments performed in triplicate. **(D)** Synergy or compensation effect exist among different fatty acid transports. The data presented are representative of three independent experiments; *n* = 3, mean ± S.E.M. **(E)** The regulative mechanism of lipid metabolism for *SLC27A1*. The data presented are representative of three independent experiments; *n* = 3, mean ± S.E.M. ^*^P < 0.05, *SLC27A1*-1220 vs. NC; ^**^P < 0.01, *SLC27A1*-1220 vs. NC.

*SLC27A1* knockdown was accomplished by RNA interference to further assess the function of *SLC27A1*. SiRNA *SLC27A1*-1220 for *SLC27A1* and NC for *GAPDH* were transfected into QM-7 cells respectively. *SLC27A1* mRNA levels decreased nearly to 50% in *SLC27A1*-1220-transfected cells compared with control NC-transfected cells (Figure 9A). To assess the effect of the *SLC27A1* knockdown in lipid metabolism, we measured the incorporation of palmitate into TG in QM-7 cells. *SLC27A1* knockdown resulted in significant increases of cellular TG concentration (4.04 ± 0.10 and 3.55 ± 0.17 mm/L, *P* < 0.05, Figure 9B), and significant decreases of cellular FFA concentration (394.27 ± 18.36 μm/L and 465.01 ± 18.21, *P* < 0.05, Figure 9C), compared with control cells. The change pattern of FFA concentration in *SLC27A1* knockdown was discrepant with that in *SLC27A1* overexpression, suggesting that *SLC27A1* may not be primary fatty acid transporter but function on lipid metabolism in QM-7 cell.

We also assessed *SLC27A4, ACSL1, GOT2*, and *CD36* mRNA levels in *SLC27A1*-1220-transfected cells compared with control NC-transfected cells by quantitative real-time PCR (Figure 9D). *SLC27A1* knockdown resulted in the down-regulated for *SLC27A4* (*P* < 0.05), but no change was observed for *ACSL1, GOT2* and *CD36*.

As well, we assessed the mRNA levels of PPARs and DEGs involved in the PPAR signaling pathway in *SLC27A1*-1220-transfected cells compared with control NC-transfected cells by quantitative real-time PCR (Figure 9E). *SLC27A1* knockdown resulted in the down-regulated for *CPT1A* (*P* < 0.01), and no changes were observed for other genes.

## Discussion

### The higher IMF content is an important factor for the WC chicken excellent meat quality

IMF content is positively correlated with flavor, tenderness, and juiciness (Fernandez et al., 1999b; Chartrin et al., 2006; Gao and Zhao, 2009; Cannata et al., 2010; Hocquette et al., 2010; Madeira et al., 2013). In pigs, increases in IMF content are accompanied by the increase in sensory tenderness (*P* = 0.001) and sensory juiciness scores (*P* = 0.017; Cannata et al., 2010). In ducks, increasing lipid levels in breast muscle promoted tenderness and flavor, with correlation coefficients of 0.43 and 0.28, respectively (Chartrin et al., 2006). IMF affects meat quality positively mainly for two reasons. First, the oxidation of IMF can dissolve the muscle fiber bundle, thus improving the tenderness and juiciness of muscles. Second, IMF contains plenty of phospholipids, the degradation of phospholipids in heating produces various kinds of volatile aromatic compounds, thus improving the flavor of muscles. The present study showed that the IMF content of WC chickens were significantly higher than that of WRR chickens at two ages (Table 1), suggesting that the higher IMF content resulted in better meat quality of WC chickens.

### Lower lipid catabolism exists in WC chicken

In the present study, we compared and analyzed the breast transcriptional profiles of WC chickens and WRR chickens of distinct genetic background, and a lot of DEGs between breeds and within breeds affecting IMF deposition were identified. These DEGs such as *ABCA1, CPT1A, LPL*, and *SLC27A1* were mostly down-regulated in WC chickens (Supplementary Table 3).

*ABCA1* (ATP-binding cassette transporter A1) functions as a cholesteral efflux pump in the cellular lipid removal pathway (Santamarina-Fojo et al., 2001; Alder-Baerens et al., 2005). *ABCA1* deficiency reduces lipid efflux, and results in lipid accumulation in the central nervous system (Hirsch-Reinshagen et al., 2004). *CPT1A* (Carnitine palmitoyl transferase I, liver) is a rate-limiting enzyme of mitochondrial fatty acid beta-oxidation (McGarry and Brown, 1997; Zammit, 2008; Akkaoui et al., 2009), and plays a prominent role in triglyceride metabolism. Overexpression of the *CPT1A* enhanced fatty acid oxidation in hepatocytes, β-Cells and muscle cells (Perdomo et al., 2004; Herrero et al., 2005; Stefanovic-Racic et al., 2008; Akkaoui et al., 2009), and decreased lipid accumulation (Stefanovic-Racic et al., 2008; Akkaoui et al., 2009). *CETP* (Cholesteryl ester transfer protein) is involved in the transfer of neutral lipids, including cholesteryl ester and triglyceride, among lipoprotein particles. Overexpressed *CETP* in SW872 cells and mice both reduced TG accumulation (Izem et al., 2015; Palmisano et al., 2016), while in chickens *CETP* was positively correlated with phospholipid accumulation (Cui et al., 2012). *LPL* (Lipoprotein lipase) has the dual functions of triglyceride hydrolase and lipoprotein uptake (Gotoda et al., 1989; Goldberg, 1996). *LPL* is an important candidate gene for chicken lipid metabolism, and the mRNA level and enzymatic activity of *LPL* was negatively correlated with IMF contents (Claire et al., 2013; Zhang X. D. et al., 2015). *SLC27A1* is involved in translocation of long-chain fatty acids (LFCAs) across the plasma membrane and subsequently lipid metabolism in skeletal muscle (García-Martínez et al., 2005; Sebastian et al., 2009; Holloway et al., 2011; Guitart et al., 2014), heart (Chiu et al., 2005), 3T3-L1 cell (Lobo et al., 2007), and 293 cell (Hatch et al., 2002).

Overexpression of the *SLC27A1* increased rate of fatty acid oxidation in heart (Chiu et al., 2005) and skeletal muscle (Nickerson et al., 2009; Sebastian et al., 2009; Holloway et al., 2011; Guitart et al., 2014), reduced TG accumulation in skeletal muscle (Guitart et al., 2014).

Moreover, *ABCA1, CPT1A, LPL*, and *SLC27A1* were all involved in the PPAR signaling pathway (Figure 7B), a well-known pathway affecting lipid metabolism (Kokta et al., 2004; Du et al., 2010), and formed a IPA® network related to lipid metabolism, molecular transport, small molecule biochemistry (Figure 5A), implying that these genes may be key genes affecting chicken IMF deposition and their down-regulated expression means that a lower lipid catabolism exists in WC chicken.

### *SLC27A1* negatively regulated lipid accumulation of chicken via CPT1A

Our RNA-seq data revealed that *SLC27A1* was lower expressed in WC.D120.B and WRR.D180.B compared with in WRR.D120.B, while the IMF content of WC.D120.B and WRR.D180.B were higher than that of WRR.D120.B (Table 1). In other words, *SLC27A1* is generally expressed lower in muscles with higher IMF contents. Our findings in cell experiments further confirmed these results. Overexpression of *SCL27A1* in QM-7 cells caused the decrease in the cellular TG content (Figure 8B), while knockdown of *SLC27A1* with RNAi resulted in the increase in the cellular TG content (Figure 9B). The reduced intramuscular TG was also observed in mouse skeletal muscle overexpressed *SLC27A1* (Guitart et al., 2014). Overall, *SLC27A1* is negatively correlated with the lipid accumulation.

Previous studies have shown that overexpressed *SLC27A1* in the skeletal muscle promoted fatty acid oxidation (Holloway et al., 2011; Guitart et al., 2014). Overexpression of *SLC27A1* increased the expression of *CPT1A* (Figure 8E), and knockdown of *SLC27A1* decreased the expression of *CPT1A* (Figure 9E), suggesting that *SLC27A1* may regulate the expression of *CPT1A* in some way. PPARs are known to be pivotal transcription factors of genes involved in lipid metabolism (Lemberger et al., 1996). Their binding to PPAR responsive element localized in the 5′-flanking region (Mascaro et al., 1998) and the second intron (Song et al., 2010) both increased *CPT1A* expression. As fatty acids and their derivatives are ligands for PPARs (Forman et al., 1995), it is possible that *SLC27A1* overexpression increases fatty acids afflux and activation, which offers ligands for binding and activating PPARs, in turn activating the PPARs-dependent gene transcription, such as *CPT1A*, a rate-limiting enzyme of mitochondrial fatty acid oxidation (McGarry and Brown, 1997; Zammit, 2008; Akkaoui et al., 2009). In addition, cellular FFA change patterns were discrepant in *SLC27A1* overexpression (Figure 8C) and knockdown (Figure 9C), indicating that *SLC27A1* plays a supplementary role in fatty acid transport, similar in rat skeletal muscle (Nickerson et al., 2009). Therefore, *SLC27A1* involves in fatty acids oxidation through collaboration with *CPT1A* (Sebastian et al., 2009), similar to *FAT/CD36* (Campbell et al., 2004; Schenk and Horowitz, 2006), but not for its transport activity across mitochondrial membrane.

Several fatty acid transporters, fatty acid translocase (FAT/CD36; Campbell et al., 2004; Schenk and Horowitz, 2006; Smith et al., 2011), plasm membrane fatty acid binding protein (FABPpm/GOT2; Clarke et al., 2004), long chain Acyl-CoA synthetase (ACSLs; Ellis et al., 2011), and fatty acid transporters (FATPs; Stahl, 2004; Kazantzis and Stahl, 2012), have been identified contributing to fatty acids uptake and oxidation. Some transporters may cooperate concertedly in fatty acids uptake, such as FABPpm and CD36 in heart and muscle (Chabowski et al., 2007), CD36 and ACSL1 in MDCK cell (Schneider et al., 2014), and ACSL1 and FATP1 in adipocytes (Richards et al., 2006). Both FATP1 and FATP4 belong to the FATP family, and their amino acid identity is 60.3% (Herrmann et al., 2001). FATP1 may involve in fatty acid uptake by hormonal regulation, while FATP4 may mediate fatty acid uptake on the basal level (Stahl et al., 2002). These fatty acid transporters are coexpressed in skeletal muscle for unknown reasons, and their functional impact with each other remains unknown. In this study, overexpression or knockdown of the *SLC27A1* could not change the expression of the *ACSL1, GOT2*, and *CD36* (Figures 8D, 9D). These findings indicate that different fatty acid transporters have distinct transcription regulatory mechanism, while *SLC27A4*, for its high homology with *SLC27A1*, was regulated similar to *SLC27A1*.

In conclusion, the present study showed that a higher IMF content resulted in a better meat quality of WC chickens. Lower lipid metabolism exists in WC chickens, the higher IMF deposition in WC chickens may be due to its lower fatty acid oxidation, and lower expression of *SLC27A1* down-regulated the fatty acid oxidation by collaboration with *CPT1A*. Moreover, these findings also indicate that reduced lipid catabolism, rather than increased lipid anabolism, contributes to chicken IMF deposition.

## Author contributions

FQ, LX, JM, WL, LZ, ZC, and SC performed experiments. FQ, LX contributed to the data analysis. FQ and XZ wrote the manuscript. FQ, LX, QN, ZL, and XZ contributed to the experimental design. All authors approved the final manuscript.

### Conflict of interest statement

The authors declare that the research was conducted in the absence of any commercial or financial relationships that could be construed as a potential conflict of interest.

## Supplementary material

### Availability of supporting data

The data discussed in this publication have been deposited in NCBI's Gene Expression Omnibus (Edgar et al., 2002) and are accessible through GEO Series accession number GSE86920 (https://www.ncbi.nlm.nih.gov/geo/query/acc.cgi?acc=GSE86920).

## Abbreviations

ABCA1: ATP-Binding Cassette Transporter A1
ACC: Acetyl-COA carboxylase
ACSL1: Long chain Acyl-CoA synthetase 1
AGPAT: 1-Acylglycerol-3-phosphate O-acyltransferase
CETP: Cholesteryl ester transfer protein
CPT1A: Carnitine palmitoyl transferase I A
DEG: differentially expressed genes
DGAT: Diacylglycerol
FABP: Fatty acid binding protein
FABPpm: Plasm membrane fatty acid binding protein
FAT/CD36: Fatty acid translocase
FBXO32: F-Box Protein 32
FDR: false discovery rate
GAPDH: Glyceraldehyde-3-phosphate dehydrogenase
GO: Gene ontology
GPAT1: Glycerol-3-phosphate acyltransferases 1
IMF: Intramuscular fat
IPA: Ingenuity Pathway Analysis
KEGG: Kyoto Encyclopedia of Genes and Genomes
LPL: Lipoprotein lipase
MUSTN1: Musculoskeletal, Embryonic Nuclear Protein 1
PPP3CA: Protein Phosphatase 3 Catalytic Subunit Alpha
QM-7 cells: Quail myoblast
qRT-PCR: quantitative real-time PCR
RNA-seq: RNA-sequencing
SLC27A1: Fatty acid transporter 1 (FATP1)
SLC27A4: Fatty acid transporter 4 (FATP4).
