# Supplementary material for: Lower Expression of SLC27A1 Enhances Intramuscular Fat Deposition in Chicken via Down-Regulated Fatty Acid Oxidation Mediated by CPT1A
Source: Front Physiol. 2017 Jun 29;8:449. doi: 10.3389/fphys.2017.00449 (PMC5489693; doi:10.3389/fphys.2017.00449)
Supplement: Supplementary file 2 [file Table2.DOCX]

**Additional file 2-Table S2. The primers used for qRT-PCR verification**

| Accession NO. | Gene symbol | Primer sequence | Anneaning temperature | Product size |
| --- | --- | --- | --- | --- |
| NM_204405.1 | ANKRD1 | F:AGTAGATATGGCTCACGG  R:ATTACTGGCAACTTATTCTC | 60°C | 243 bp |
| XM_423102.3 | PPP1R3C | F:CGAGCCAAGAAGCGAGTT  R:AGCTGAGGGACGAGGAAA | 61°C | 192 bp |
| NM_001034814.1 | CETP | F: CGCTGCTGTCTGAATCCC  R: TAGCCACAGAATCATTGTAG | 56°C | 166 bp |
| NM_001031258.2 | PRKAG3 | F:AGTTCCTCCACATCTTCGG  R:GACACGGCGGTCCACAAA | 55°C | 155 bp |
| NM_001039602.1 | SLC27A1 | F:TGCCTTCCGCTCTACCAC  R:TCAACCCGTTTGCCCACT | 59°C | 239 bp |
| XM_424759.4 | PIK3R1 | F:CTAAGCCACCGAAACCTA  R:AAGCCGTATTTCCCATCT | 59°C | 250 bp |
| NM_205282.1 | LPL | F:GTACAGTCTGGGTGCTCAT  R:GGAAACCTCCACCATTAG | 57°C | 248 bp |
| NM_001030956.1 | FBXO32 | F:AGTGCTCAGCGAAGACCG  R:TACTGCTCCTTCCGTGGG | 56°C | 159 bp |
| NM_001012898.1 | CPT1A | F:ATGGCTGGATGTTTGCTG  R:TCATAAGTGGCCGGACTG | 63°C | 186 bp |
| NM_204450.2 | CREB1 | F:CCAGACGACCCTCCTACA  R:TGAATTGCTCCTCCTTGC | 59°C | 190 bp |
| NM_001006457.1 | ABCA1 | F:TCCTCTGGCTTAGACTTGA  R:CTCGTAGTTGTATTCGGTAA | 55°C | 169 bp |
| NM_001177329.1 | LASP1 | F:CAGGAACTCCCAGGAAAGC  R:CGTTGTAATCATAGACGGCAC | 59°C | 206 bp |
| NM_001277701.1 | FABP6 | F:ATGGAGACAATGGGTGGTA  R:AGGTGGCCTTAGGAACAG | 56°C | 182 bp |
| NM_001012696.1 | PIK3CD | F:CGAGCCAAGAAGCGAGTT  R:AGCTGAGGGACGAGGAAA | 61°C | 192 bp |
| NM_001007839.1 | YWHAH | F:AGATTATTACCGCTATTTGG  R:CAGGAGCATTCTGGATTT | 53°C | 188 bp |
| NM_204871.1 | IL6ST | F:GTGGGTAGAAGCAGCAAA  R:ACTGATCCTGTAGCGAAT | 53°C | 202 bp |
| NM_213580.2 | MUSTN1 | F:TGTGAACAAATGGGCTCT  R:TTGGGTATCTGTGGCTATG | 55°C | 207 bp |
| NM_205274.1 | MYH11 | F:GGCTTCAACAACTACACCT  R:ACAGACGAAACAACCCTC | 53°C | 152 bp |
| XM_004935966.1 | PPP3CA | F:AGATGCACCAGTCACAGT  R:TGATTCCCACGAAGTAAA | 57°C | 210 bp |
| NM_204396.1 | CIP1 | F:GGGCAGACCACCATCAAA  R:GGGAACTACAGACTCGGCATT | 63°C | 185 bp |
