# Supplementary material for: Lower Expression of SLC27A1 Enhances Intramuscular Fat Deposition in Chicken via Down-Regulated Fatty Acid Oxidation Mediated by CPT1A
Source: Front Physiol. 2017 Jun 29;8:449. doi: 10.3389/fphys.2017.00449 (PMC5489693; doi:10.3389/fphys.2017.00449)
Supplement: Supplementary file 3 [file Table3.DOCX]

**Additional file 3-Table S3. Key DEGs related to chicken IMF deposition**

| ID | Gene Symble | Gene Title | ↑ or ↓ |
| --- | --- | --- | --- |
| WRR.D120.B-VS-WC.D120.B | | | |
|  | ABCC1 | ATP-binding cassette, sub-family C, member 1 | ↑ |
|  | ACACB | PREDICTED: acetyl-CoA carboxylase beta | ↓ |
|  | AGMO | PREDICTED: alkylglycerol monooxygenase | ↓ |
|  | AGPAT2 | PREDICTED: 1-acylglycerol-3-phosphate O-acyltransferase 2 (lysophosphatidic acid acyltransferase, beta) | ↓ |
|  | AKR1B1L | aldo-keto reductase family 1, member B1-like (aldose reductase) | ↑ |
|  | ANKRD1 | ankyrin repeat domain 1 (cardiac muscle) | ↑ |
|  | CETP | cholesteryl ester transfer protein, plasma | ↑ |
|  | CPT1A | carnitine palmitoyltransferase 1A (liver) | ↓ |
|  | CREB1 | cAMP responsive element binding protein 1 | ↓ |
|  | DKK3 | dickkopf homolog 3 (Xenopus laevis) | ↑ |
|  | FNBP1 | formin binding protein 1 | ↑ |
|  | GPX1 | glutathione peroxidase 1 | ↓ |
|  | ITGAV | integrin, alpha V | ↓ |
|  | KIT | v-kit Hardy-Zuckerman 4 feline sarcoma viral oncogene homolog | ↓ |
|  | LPL | lipoprotein lipase | ↓ |
|  | MBOAT2 | membrane bound O-acyltransferase domain containing 2 | ↓ |
|  | NCOA2 | PREDICTED: nuclear receptor coactivator 2 | ↓ |
|  | PDGFRA | platelet-derived growth factor receptor, alpha polypeptide | ↓ |
|  | PIK3R1 | phosphoinositide-3-kinase, regulatory subunit 1 (alpha) | ↓ |
|  | PLA2G4B | PREDICTED: phospholipase A2, group IVB (cytosolic) | ↓ |
|  | PLA2G7 | phospholipase A2, group VII (platelet-activating factor acetylhydrolase, plasma) | ↓ |
|  | PLA2G12A | PREDICTED: phospholipase A2, group XIIA | ↑ |
|  | PNPLA2 | patatin-like phospholipase domain containing 2 | ↓ |
|  | PRKAG3 | protein kinase, AMP-activated, gamma 3 non-catalytic subunit | ↑ |
|  | SAMD8 | PREDICTED: sterile alpha motif domain containing 8 | ↓ |
|  | SLC27A1 | solute carrier family 27 (fatty acid transporter), member 1 | ↓ |
| WRR.D180.B-VS-WC.D180.B | | | |
|  | CAT | catalase | ↓ |
|  | FABP6 | fatty acid binding protein 6, ileal | ↓ |
|  | LRP1 | low density lipoprotein receptor-related protein 1 | ↓ |
|  | PIK3CD | phosphoinositide-3-kinase, catalytic, delta polypeptide | ↓ |
|  | TOP2A | topoisomerase (DNA) II alpha 170kDa | ↓ |
|  | YWHAH | tyrosine 3-monooxygenase/tryptophan 5-monooxygenase activation protein, eta polypeptide | ↑ |
| Both WRR.D120.B-VS-WC.D120.B and WRR.D180.B-VS-WC.D180.B | | | |
|  | ABCA1 | ATP-binding cassette, sub-family A (ABC1), member 1 | ↓ |
| WRR.D120.B-VS-WRR.D180.B | | | |
|  | ANKRD1 | Ankyrin repeat domain 1 (cardiacmuscle) | ↑ |
|  | DECR1 | 2,4-dienoyl CoA reductase 1, mitochondrial | ↓ |
|  | ESR1 | Estrogen receptor 1 | ↓ |
|  | PLA2G4B | Phospholipase A2, group IVB (cytosolic) | ↓ |
|  | SLC27A1 | Solute carrier family 27 (fatty acid transporter), member1 | ↓ |
|  | SRD5A2 | PREDICTED: steroid-5-alpha-reductase, alphapolypeptide 2 (3-oxo-5alpha-steroiddelta4-dehydrogenase alpha 2) | ↓ |
|  | YWHAH | Tyrosine 3-monooxygenase/tryptophan 5-monooxygenase activation protein, etapolypeptide | ↑ |

Note:↑, up-regulated in WC; ↓, down-regulated in WC.
