# Supplementary material for: Lower Expression of SLC27A1 Enhances Intramuscular Fat Deposition in Chicken via Down-Regulated Fatty Acid Oxidation Mediated by CPT1A
Source: Front Physiol. 2017 Jun 29;8:449. doi: 10.3389/fphys.2017.00449 (PMC5489693; doi:10.3389/fphys.2017.00449)
Supplement: Supplementary file 4 [file Table4.DOCX]

**Additional file 4- Table S4. IPA interaction network of DEGs**

| ID | Top diseases and functions | Focus molecules | Molecules in network |
| --- | --- | --- | --- |
| WRR.D120.B-VS-WC.D120.B | | | |
| 1 | Post-translational modification, infectious Disease, organismal injury and abnormalities | 26 | ↓AGPAT2, ↑ASB13, ↑CDC34, ↓CNOT1, Dynamin, E2f, ↓EFEMP1, ↓ENOX2, ↓EPAS1, ↑FBXO32, ↓FXYD6, ↑GLS, ↑KLF11, ↑MED7, ↑MED18, MHC CLASSⅠ(family), ↑NEDD4, ↑PRKAG3, Ras, ↓RERE, RNA polymerase Ⅱ, Rnr, ↓RPL12, ↑RPS5A, Sapk, ↑SBDS, ↑SDHB, ↑SEPT5, ↓SEPT9, Septin, ↑TCEB1, ↓TRIM47, ↑TSG101, Ubiquitin, ↑WSB2 |
| 2 | Organismal injury and abnormalities, renal and urological disease, cardiovascular disease | 25 | ↑ABCC1, ADCY, ↑AR, ↓ATF7IP, ATPase, Camk, ↓CAMK1D, ↓CBFA2T3, ↓CREB1, ↑CTNNBIT1, ↓CYP2D6, ↑EAF1, Cytochrome bc1, ↑DCUN1D5, ↓GNAL, ↓HDAC7, ↓HSPD1, ↑ISCU, Mitochondrial complex 1, ↓MKL2, ↓ MMRN1, ↓MYH9, NADH dehydrogenase, ↑NDUFA11, ↑NDUFB1, ↑NDUFB2, Pka, ↑RCHY1, TSH, ↑UQCR10, ↑UQCRFS1, ↑UQCRH, ↓ZNF703 |
| 3 | Cell-to-cell signaling and interaction, cellular compromise, cellular movement | 22 | ↓ANTXR2, ↓CEP350, Collagen type XⅧ, ↓CTTNBP2 , ↓DAPK1, ↓EFNB1, ↓EPHA3, ↓EPHB1, ERK, ↓FBLN2, Fgf, ↑FGF6, Fgfr, Hspg, Integrin, ↓LAMA1, Laminin, Laminin1, ↓LRIG1, ↑LRRFIP1, ↓MAPKAPK3, ↓MKNK2, ↑MYF6, NCK, ↓PDE10A, ↓PEAK1, Ppp2c, ↓RAB2A, Rap1, Rsk, ↓SDC1, ↓TLN1, Troponin t, ↓VCL, ↓WDR44 |
| 4 | Cellular development, developmental disorder, digestive system development and function | 22 | 19S proteasome, ↑ADRM1, ↓C9orf3, ↑CAPZA2, ↓CCAR1, ↑CKMT2, creatine kinase, Ctnna, ↓CTNNA1, Dishevelled, ↓EGFR, Frizzled, ↑IARS, ↑IDH3A, ↓JAG1, Lpa receptor, MAP1LC3, MEF2, NFkB(family), Notch, ↓NOTCH2, PI3K(family), PI3K p85, ↓PNPLA2, ↑PPIF, ↑PSMD4, ↑PSMD12, ↓RANBP1, ↑RPS27L, ↓SEPP1, ↓SKI, Smad, ↓SMAD1, ↑TIMM17A, ↑TIMM8A |
| 5 | Energy production, nucleic acid metabolism, small molecule biochemistry | 23 | Adenosin-tetraphosphatase, Akt, ↓ARHGAP15, ATP synthase, ↑ATP5C1, ↑ATP5H, ↑ATP5J, ↑ATP5L,↑ATP6V0C, Cofilin,↑ATP6V1E1, ↓CDC42BPA, ↑COPS8, CYP19, F0 ATP synthase, ↑FBXW4, ↓FHOD3, ↑FNBP1, H+ -exporting ATPase, ↑LAMTOR2, Limk, Mlc, ↓MTSS1, Pak, PDGF(family), ↓PDGFD, ↓PHIP, ↓PIK3IP1, ↓PLXNB1, ↓PREX2, ↓PTRF, Rock, ↓ROCK1, ↑SEMA7A, ↑SLC35B2 |
| 6 | Cellular compromise, Cellular function and maintenance, post-translational modification | 21 | ↓ACTN1, CD8, ↑DKK3, DNAJ, ↓DNAJA4, ↑DNAJB2, ↑DNAJB9, Hdac, ↓HLA-DRA, HSP, Hsp70, Hsp90, Hsp22/Hsp40/Hsp90, ↓HSPA8, ↓HSPA4L, ↑HSPB7, ↓HSPB8, ↓HSPH1, Jnk, ↑KLHL38, ↓MAP4K5, MHC, MIR124, MHC Class Ⅱ(complex), MKK3/6, ↓MYO1C, ↑MYOT, ↓NFAT5, NFAT(complex), ↓NFATC3, ↓OSR1, ↓PCDH1, ↑PPM1A, ↑SUNPO2, Tlr |
| 7 | Cancer, endocrine system disorders, gastrointestinal disease | 21 | ↓AMPD3, ↓ASAP1, ↓AXIN1, c-Src, ↓CBL, Ctbp, ↓CXXC5, ↓EPB41L3, ↓FLT1, GTPase, Hedgehog, ↑HIPK2, JAK, ↓KDR, ↑KIFC1, ↓KIT, ↓LYVE1, ↓MDFIC, ↓MSC, ↑MYH2, ↓PDGFRA, Pias, PLC gamma, ↓PRC1, ↓PTCH1, PTPase, ↓PTPRB, ↓PTPRC, ↓PTPRG, Sod, SRC(family), TCF, tyrosine kinase, Vegf, Wnt |
| 8 | Cancer, gastrointestinal disease, organismal injury and abnormalities | 20 | ↓ADD3, ↑AMPD1, ↓ANXA2, ↓ANXA5, Caveolin, ↓COLEC12, Collagen type Ⅲ, Collagen type Ⅳ, Collagen(s), Cyclin D, Cyclin E, Fcer1, ↓FHL2, Hsp27, ↓LAMP2, LDL, ↓MMP16, ↓MYH11, Myosin, ↓NPR3, P38 MARK, ↓P4HB, Pdgf(complex), PDGF BB, PLA2, ↓PLA2G7, ↓PLA2G12A, ↓PLA2G4B, ↓PLA2R1, ↓SERPINH1, ↓SESN1, ↑SNRPG, ↓TNNC1, ↑TNNC2, trypsin |
| 9 | Lipid metabolism, molecular transport, small molecule biochemistry | 20 | ↓ABCA1, ↓ACACB, ↑Akr1b7, ↑ANKRD1, ↓ASXL2, ↓CBX7, ↓CCDC50, ↑CETP, ↓CLIP1, CPT1, ↓CPT1A, ↓DAP, HDL, GC-GCR dimmer, ↓HIVEP2, JUN/JUNB/JUND, ↓KCNJ4, ↓LPL, ↓MYPN, N-cor, ↓NCOA2,Nr1h, ↓OTUD7B, NFkB(complex), PPARα-RXAα, PRKAC, Rar, Rxr, ↓SLC27A1, ↓STK10, thyroid hormone receptor, VitaminD3-VDR-RXR, VLDL-cholesterol, ↑ZFAND5 |
| 10 | Gene expression, cardiovascular disease, cell cycle | 20 | ↓BRD4, ↑C19orf70, Cbp/p300, Cg, ↓CHD4, Cyclin A, estrogen receptor, ↓FAM20C, ↓FOXK2, FSH, ↑FTH1, ↓GDF10, Growth hormone, HDL-cholesterol, hemoglobin, HISTONE, Histone h3, Histone h4, ↓IGF1R, ↓JMJD6, LDL- cholesterol, Lh, ↓MTR, ↓NFYA, ↓PPP1R15B, ↑RPL39, SAA, ↓SOX8, STAT5a/b, ↑SUV39H1, ↓TGFBR2, ↓TRIM24, ↓TSC22D1, ↓UBN1, ↓VNN1 |
| 11 | Cellular movement, cardiovascular system development and function, Cancer | 20 | ↓CDH5, ↓CDH11, Collagen alpha1, Collagen type Ⅱ, Cpla2, ↓CTNNAL1, Ecm, EGFR/PDGFR/IGFR,  ↓ENG, ERK1/2, ETS, Fibrin, ↓FIGF, ↓FATL3, Igf, ↓IGFBP2, ↓IGFBP4, ↓ITGA6, ↓ITGAV,  ↓MGAT3, ↑MYH13,Neuropilin, ↓PCOLCE2, ↓PLXDC2, ↑PLXNB2, ↓PLXND1， Secretase gamma, ↓SEMA3F, SMAD1/5, Smad1/5/8, Smad2/3, ↓TGFB3, TGFBR, ↓TRIM2, ↓VEGFC |
| 12 | Cell-to-cell signaling and interaction, cellular assembly and organization, post-translational modification | 17 | Actin, ↓ACTN4, Alpha actin, Alpha catenin, Arp2/3, Cadherin, Calpain, ↓CAPN6, ↓CAST, ↓CDH2, Collagen type Ⅰ, ↓CTNNA2, ↓DIAPH2, ENaC, Erm, F Actin, Filamin, Integrin β, ↓IQGAP1, ↓LASP1,↓KLHL40,↑LMOD3, N-cadherin, ↓PCDH19, PI3K(complex), ↓PIK3R1, Profilin, ↓SERPINE2, ↓TGP1, ↑SLC20A2, sphingomyelinase, SYK/ZAP, ↓TGP2, ↑TPP2, VAV |
| 13 | Cell signaling, molecular transport, vitamin and mineral metabolism | 18 | 14-3-3, ADRB, ↑ANKH, ↓AQP4, ↓ASPH*, ↑CACNA1S, ↑CACNG1, Calcineurin protein(s), CaMKⅡ, CK1, ↑FAM214A, ↓FLNB, Glycogen synthase, growth factor receptor, ↓HOMER3, ITPR, ↓ITPR3, L-type calcium channel, MAP2K1/2, Nfat(family), NMDA receptor, phosphatase, Pkc(s), ↓PKD2, Pkg, PP2A, Ptk,↑PPM1J, ↑PPP1R3C, ↑PVALB, ↓PYGL, SAMD4A*, ↓SETBP1, ↑TRPC3, ↓UPF1 |
| 14 | Humoral immune response, protein synthesis, gastrointestinal disease | 16 | AcHR, ALT, ↓ARNTL, ↑B2M, BCR(complex), ↓CD69, Cdc2, ↓CFH, Fc gamma receptor, ↓FOXP1, ↓GADD45A, ↓GNAI2, GOT, ↓GPX1, IFN alpha/beta, Ifnar, Iga, Ige, IgG, IgG1, IgG2a, IgG2b, ↓IGJ, Igm, ↑IL11RA, IL12（complex）, ↓IL6ST, Immunoglobulin, ↓MAN2A1, Mapk, mediator, ↓MYD88, ↓NFIL3, ↓PPL, ↑RAPSN |
| 15 | Connective tissue disorders, developmental disorder, hereditary disorder | 16 | ↓AKAP2, AKAP11, ↓ASPM, CEP135, CTDP1, EHD2, EPN2, ↑EPN3, Epsin, ESYT1, ↓ESYT2, ↑FAM08A, ↑FUNDC1, ↓GPBP1, LAPTM5, ↑MRPS12, ↑MZT1, MZT2A, ↓N4BP2, ↑PDCD2L, RALGAPA1, ↓RALGAPB, REPS1, ↓RSF1, ↓SASS6, SIX6, SLC25A46, SMYD2, STIL, ↓TLE4, TUBGCP5, TUBGCP6, UBC, XRCC3, ↑ZNHIT3 |
| 16 | Cell cycle, developmental disorder, hereditary disorder | 16 | ARMC1, ↑C11orf31, ↑C12orf5, CEP57, CKAP5, ↑COQ10B, CREB3L1, ↓CRIM1, ↑DDO, DOCK2, EVL, FYB, GOLGA7, HAUS2, ↓HEBP2, ↑HINT3, LAMTOR5, LRPPRC, MAGEA12, ↑MFSD5, PDCD6,↑MTFR1L, PEX5, PEX19, RPL11, RREB1, ↑SLC39A3, TENM2, ↓TENM3, ↓TMEM131, ↓TMEM164, ↑TMEM167B, UBC, ↑ZDHHC9, ↓ZRSR2 |
| WRR.D180.B-VS-WC.D180.B | | | |
| 1 | Connective tissue developmental and function, organ morphology, Organismal development | 20 | ↓FSTL1, ↓SIM2, ↓OGN, ↓SERPINE2, ↓GADD45A, ↓IL6ST, ↑CNTFR, ↓AQP4, ↑FABP6, ↓CCDC50, LDL, ↓ FLNB, ↓CAT, ↓MICAL2, ↑OUTD1, ↑PRKCH, Ubiquitin, ↓MSC, ↓ASB2, ↑ PON2 |
| 2 | Cancer, organismal injury and abnormalities, endocrine system disorders | 20 | ↑PSMD3, ↑RPL39, ↑SPRY4, ↓PTPN21, ↑DUSP8, ↓TRIM2, ↓PTPRB, ↑ LIMS1, ↓PIK3CD, ↓TGFBR3, ↓ NRP1, ↓PTN, ↓ABCA1, ↓PDGFRB, ↓FLT1, ↓LRP, ↓FLT4, ↓COL4A1, ↓ADD3, Collagen type Ⅳ |
| 3 | Nerve system development and function, tissue morphology, cancer | 17 | ↓NDE1, ↑TMOD4, ↑HSF2BP, ESR1, ↓ ZNF366, ↑POMP, ↓KY, ↑ASB15, ↓FHOD3, ↓TGFBR3, ↓FREM2, ↑FAM110A, ↑ZP1, ↑ PITX3, ↓ABI3BP, ↓MFAP5, ↓SLC38A1, |
| 4 | Embryonic development, organ development, organismal development | 17 | ↓COL28A1, ↓COL12A1, ↓COL4A2, ↓STAB1, ↓SPARC, ↑WNT9A, ↑WNT5B, ↓THY1, ↑BMP7, ↓MMP2, ↓ CDH5, ↓ITGA6, ↑LINGO1, ↓PREX2, ↓CCDC80, ↓SERPINB10,Collagen |
| 5 | Dermatological diseases and conditions, developmental disorder, heredity disorder | 15 | ↑DUSP13, ↓SLC43A2, ↑R3HDM4, ↓DOPEY2, UBC, ↓DCBLD1, ↑HDDC2, ↑KIAA1161, ↑CCDC134, ↑MNT5B, ↓THSD7B, ↓VPS13A, ↓MXRA5, ↓LONRF3, ↑MYADML2 |

Note: ↓, Down-regulated in WC; ↑, Up-regulated in WC.
