# Supplementary material for: Lower Expression of SLC27A1 Enhances Intramuscular Fat Deposition in Chicken via Down-Regulated Fatty Acid Oxidation Mediated by CPT1A
Source: Front Physiol. 2017 Jun 29;8:449. doi: 10.3389/fphys.2017.00449 (PMC5489693; doi:10.3389/fphys.2017.00449)
Supplement: Supplementary file 5 [file Table5.DOCX]

**Additional file 5- Table S5. The primers used for function verification of *SLC27A1***

| Accession NO. | Gene symbol | Primer sequence | Anneaning temperature | Product size |
| --- | --- | --- | --- | --- |
| NM_001039602.1 | SLC27A1 | F:TGCCTTCCGCTCTACCAC  R:TCAACCCGTTTGCCCACT | 59°C | 239 bp |
| NM_001030731.1 | CD36 | F:TGTCATTGGTGCTGTGCT  R:TGTAAGGTCCTCTTTGTTC | 61°C | 236 bp |
| NM_001012578.1 | ACSL1 | F:AGGTAGAGGGTGAAGAGG  R:GTGAAGTAATGCAGAGCC | 55°C | 230 bp |
| NM_205523.1 | GOT2 | F:AGGGTATTTCTGGGACTGG  R:GGTCGTAGTAGCGGTAAGC | 60°C | 162 bp |
| XM_415504.4 | SLC27A4 | F:CCCGATGATGTGATGTATGA  R:CTCCTGGTAGGGCTGGTT | 55°C | 213 bp |
| NM_001001464.1 | PPARA | F:ACAGACACCCTTTCACCA  R:GTTTCAATCGGATGGTTC | 55°C | 223 bp |
| NM_204728.1 | PPARD | F: ACTGCCGCTTCCAGAAAT  R: TAGGCGTTGTAGATGTGCT | 55°C | 179 bp |
| NM_001001460.1 | PPARG | F:TCCTTCCCTCTGACCAAA  R:AATCTCCTGCACTGCCTC | 59°C | 216 bp |
| NM_001012898.1 | CPT1A | F:ATGGCTGGATGTTTGCTG  R:TCATAAGTGGCCGGACTG | 63°C | 186 bp |
| NM_205282.1 | LPL | F:GTACAGTCTGGGTGCTCAT  R:GGAAACCTCCACCATTAG | 57°C | 248 bp |
| NM_001034814.1 | CETP | F: CGCTGCTGTCTGAATCCC  R: AGCCACAGAATCATTGTAG | 56°C | 166 bp |
| NM_001006457.1 | ABCA1 | F:TCCTCTGGCTTAGACTTGA  R:CTCGTAGTTGTATTCGGTAA | 55°C | 169 bp |
